# Prevalence of Poor Mental Health Days and Adverse Childhood Experience Reporting in U.S. Adults Before and After COVID-19

**DOI:** 10.1007/s10597-022-01001-0

**Published:** 2022-07-13

**Authors:** Julie M. Kapp, Lada Micheas, Shannon Holmes, Melissa Stormont, Wendy M. Reinke

**Affiliations:** 1 Department of Health Management and Informatics, School of Medicine, University of Missouri, CE 717 CS&E Building, One Hospital Drive, Columbia, MO 65212 USA; 2 Harry S Truman School of Public Affairs, University of Missouri, Columbia, MO 65212 USA; 3 Department of Statistics, Social Science Statistics Center, University of Missouri, Columbia, MO 65211 USA; 4 Department of Educational, School & Counseling Psychology, College of Education & Human Development, Missouri Prevention Science Institute, University of Missouri, 16 Hill Hall, Columbia, MO 65211 USA; 5 Department of Special Education, College of Education & Human Development, Missouri Prevention Science Institute, University of Missouri, 311N Townsend Hall, Columbia, MO 65211 USA; 6 Department of Educational, School & Counseling Psychology, College of Education & Human Development, Missouri Prevention Science Institute, University of Missouri, Columbia, MO 65211 USA

**Keywords:** COVID-19, Coronavirus, Adverse childhood experiences, Mental health, Health status disparities

## Abstract

This is the first study of US adults to examine change in the prevalence of psychological burden (i.e., self-reported poor mental health days in the past 30 days, and ACEs recollections) before compared to after COVID-19 started. We analyzed the prevalence of self-reported poor mental health days, and ACEs recollections from 17 states using the Behavioral Risk Factor Surveillance System. Adjusted models identified an increase in prevalence from before compared to after COVID-19 onset in those married or partnered reporting 48% more poor mental health days in the past 30 days; persons of color reporting living with anyone with mental illness during childhood by 73% and reporting more ACEs by 35%; those employed or self-employed reporting childhood sexual abuse by 45%. This ecological-level analysis revealed population-level changes in psychological well-being reporting of U.S. adults from before compared to after the pandemic onset.

## Introduction

On January 9, 2020, the World Health Organization (WHO) convened their first teleconference on COVID-19 (World Health Organization, 2021); March 11, 2020, they declared the outbreak a pandemic (World Health Organization, 2021); and only seven days later, they published guidance on mental health and psychological considerations (World Health Organization, 2021). Despite thousands of publications catalogued in PubMed on mental health during COVID-19, studies on the prevalence of mental health conditions in US adults during this time period are limited. Examining the psychological health of the US population before and after the COVID-19 pandemic began provides critical insight into the population burden.

Available research for US adults suggests significant changes in mental health or related behaviors during the pandemic. Adults in 2020 were considerably more likely to screen positive for mood disorders compared to 2019 (Twenge & Joiner, 2020). US adults reported increases in the number of drinking days, with sustained increases among males, White participants, and older adults (Nordeck et al., 2021). The proportion of US adults with high levels of anxiety symptoms increased significantly from 8% in 2019 to 21% in April 2020, declined to 11% in May 2020, and plateaued above 2019 levels until December 2020 (Daly & Robinson, 2021). The prevalence of depression symptoms was more than three times higher during the COVID-19 pandemic compared to before, with lower income, having less than $5,000 in savings, and having exposure to more stressors associated with greater risk of depression symptoms (Ettman et al., 2020b). Other sources of psychosocial stress included: personal health or finances (Holingue, Badillo-Goicoechea, et al., 2020; Holingue, Badillo-Goicoechea, et al., 2020; Holingue, Kalb, et al., 2020; Holingue, Kalb, et al., 2020); family health (McKnight-Eily et al., 2021); feelings of isolation (McKnight-Eily et al., 2021); infecting others (Holingue, Badillo-Goicoechea, et al., 2020; Holingue, Kalb, et al., 2020; McKnight-Eily et al., 2021); worry about persons dying (McKnight-Eily et al., 2021); workplace exposure (McKnight-Eily et al., 2021); being blamed for spreading COVID-19 (McKnight-Eily et al., 2021); major changes to personal life (Holingue et al., 2020a; Holingue, Kalb, et al., 2020); and searching online or using social media to post about coronavirus (Holingue, et al., 2020a, 2020b). Mental health symptoms elevated since before the pandemic are not transient but persist (Czeisler et al., 2021).

The prevalence of current depression, suicidal ideation, and increased or newly initiated substance use was higher in Hispanic respondents than other racial/ethnic groups (McKnight-Eily et al., 2021). Lee and Singh (Lee & Singh, 2021) found a higher odds of having fair or poor health status and serious depression for adults with less than high school education, lower income, and renters, compared to those with higher education, higher income, and homeowners. Moreover, the odds of experiencing fair or poor health was higher for racial and ethnic minorities compared to non-Hispanic Whites, while the odds of having serious depression was higher for non-Hispanic Whites and non-Hispanic others, compared to non-Hispanic Blacks, Hispanics, and non-Hispanic Asians (Lee & Singh, 2021). Populations with low assets bear a greater burden of mental illness during the COVID-19 pandemic (Ettman et al., 2020a).

In addition to mental health, another important indicator of psychological burden is adverse childhood experiences (ACEs). ACEs are well-established as clinically meaningful self-reported indicators of trauma history and well-being. ACEs are common in the U.S., with an estimated 61% of adults experiencing at least one ACE category, and 16% experiencing ≥ 4 categories (Merrick et al., 2019). Pre-pandemic studies report population subgroups more likely to experience more ACE exposures include women and persons of color (Merrick et al., 2018, 2019). ACEs increase the risk of obesity, chronic diseases, and mental disorders in adults (Felitti et al., 1998; Merrick et al., 2019) through coping behaviors like overeating, smoking, drug use, and promiscuity, as well as chronic stress (Felitti, 2009). The economic and social costs of ACEs total hundreds of billions of dollars each year (Centers for Disease Control & Prevention, 2020).

The empirical literature on the prevalence of ACEs during COVID-19 is even more limited. At the time of this writing, PubMed included only 32 search results on ACEs, COVID-19, and prevalence. Most articles were commentaries, letters to the editor, or studies of populations outside the U.S. Of relevant literature, those with ACEs are more likely to develop anxiety disorders and perceive a greater threat from COVID-19 (Kalia et al., 2020).

Given the scarce literature, we ask the following research questions:

- Is there a measurable change in the prevalence of psychological burden (defined as self-reported poor mental health days in the past 30 days, and ACEs recollections) among U.S. adults from before to after COVID-19 began? We hypothesized that yes, there is a difference.
- If so, by how much?
- If so, are there differences by population subgroups?

## Methods

### Study Design

This is an ecological-level analysis of the 2019 Behavioral Risk Factor Surveillance System (BRFSS) data across two time periods. The BRFSS is a cross-sectional telephone survey conducted by US state health departments monthly over landline and cellular telephones using a standardized questionnaire, with assistance from the CDC (Centers for Disease Control & Prevention, 2013; National Center for Chronic Disease Prevention and Health Promotion, 2020). The BRFSS Data User Guide (Centers for Disease Control & Prevention, 2013) details the methodology for standardized data collection across states, as well as the weighting methodology used to mitigate sampling bias.

### Setting and Participants

**Fig. 1:**
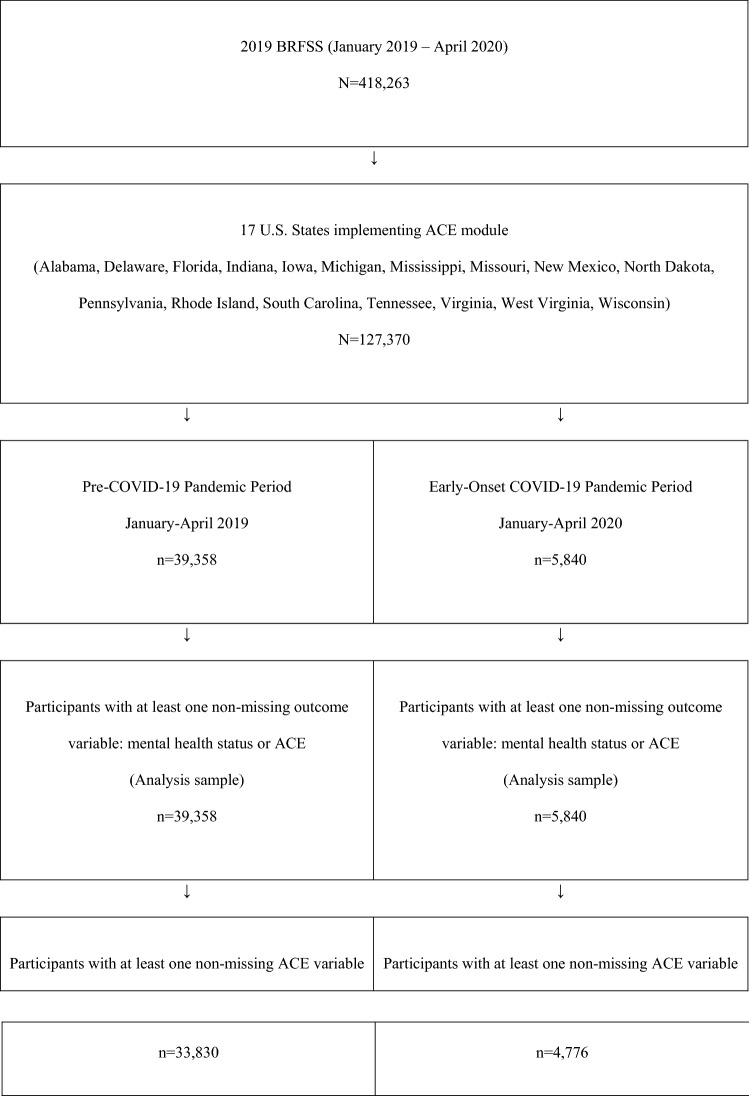
Sample selection flow diagram

The BRFSS captures population-based prevalence data from non-institutionalized U.S. adult residents ages 18 and older related to their risk behaviors, health conditions, and health practices. We used the “2019 BRFSS Questionnaire Data (Combined Landline Telephone and Cellular Telephone)” dataset for states that used common modules on both the landline survey and cellphone survey (National Center for Chronic Disease Prevention and Health Promotion, 2020). Our sample selection, including the 17 states implementing the common version of the ACEs module, is described in Fig. 1.

### Measurement

Our exposure of interest is before compared to after COVID-19 began. We created a variable from the survey participants’ response month to indicate the pre-COVID-19 pandemic period of January through April 2019 versus the early-onset COVID-19 pandemic period of January through April 2020. This decision is based on: (1) the heightened stress of the early phase of the pandemic, and (2) data available in the 2019 BRFSS.

Our outcome of interest is the prevalence of reported psychological burden among U.S. residents. First, we define psychological burden as self-reported poor mental health days. Poor mental health days are captured by the question “Now thinking about your mental health, which includes stress, depression, and problems with emotions, for how many days during the past 30 days was your mental health not good?” We reported the CDC’s response categories of zero, 1–13, or 14+ days (National Center for Chronic Disease Prevention and Health Promotion, 2020). Second, we define psychological burden as ACEs reflections. The ACEs module includes 11 questions intended to reflect respondents’ recollections of life before 18 years of age (National Center for Chronic Disease Prevention and Health Promotion, 2020). These questions can be combined into eight types of exposures across three areas (Ford et al., 2014; Merrick et al., 2019):

Household dysfunction:

1. Mental illness: “Did you live with anyone who was depressed, mentally ill, or suicidal?”;
2. Substance abuse: “Did you live with anyone who was a problem drinker or alcoholic?”; “Did you live with anyone who used illegal street drugs or who abused prescription medications?”;
3. Incarcerated household member: “Did you live with anyone who served time or was sentenced to serve time in a prison, jail, or other correctional facility?”;
4. Parental separation or divorce: “Were your parents separated or divorced?” (parents not married set to missing);
  Emotional/Physical Abuse:
5. Intimate partner violence: “How often did your parents or adults in your home ever slap, hit, kick, punch or beat each other up?”
6. Physical abuse: “Not including spanking, (before age 18), how often did a parent or adult in your home ever hit, beat, kick, or physically hurt you in any way?”
7. Emotional abuse: “How often did a parent or adult in your home ever swear at you, insult you, or put you down?”
  Sexual abuse:
8. Sexual abuse: “How often did anyone at least 5 years older than you or an adult, ever touch you sexually?”; “How often did anyone at least 5 years older than you or an adult, try to make you touch them sexually?”; “How often did anyone at least 5 years older than you or an adult, force you to have sex?”

Following from the literature, we calculated the respondents’ ACE score as the sum of exposures (yes/no or ever/never) ranging from 0 to 8 (Ford et al., 2014; Merrick et al., 2019; Waehrer et al., 2020). We then categorized the “sum of ACEs” into zero, one, two to three, or four or more exposure groups (Merrick et al., 2019; Waehrer et al., 2020).

Identifying a change in population-level prevalence from before to after COVID-19 began in reflections on childhood exposures may signal an historical cultural shift in the population’s perceived psychological threats and experiences. ACEs have high test–retest reliability (Dube et al., 2004; Frampton et al., 2018; Karatekin & Hill, 2019; Mersky et al., 2017), even when considering symptoms of depression and recall of negative memories (Frampton et al., 2018). Test–retest reliability is also acceptable for the sum of ACEs over 6 to 20 months, though with variable results for individual ACEs (Karatekin & Hill, 2019). If adults do misremember ACEs, it tends to be an underestimate (Frampton et al., 2018). Bethell et al. (Bethell et al., 2017) noted that the “ACEs assessment is not intended to objectively document the occurrence of events, but to assess recollection of experiences of such events…perceived experiences drive many of the effects of concern.”

### Demographic Variables

We characterized our sample using the following variables we identified as theoretically relevant, including: respondent sex, age group, race/ethnicity ((Non-Hispanic White vs other (non-Hispanic Black, non-Hispanic Other, Hispanic), hereafter referred to as persons of color (POC)), education, employment status, annual household income, and whether the respondent owns versus rents their home or there is another arrangement (e.g. group home, staying with friends or family without paying rent). We included metropolitan status (rural or urban); married or partnered (versus divorced, widowed, separated, never married); the number of adults and children in the household; health care coverage (yes/no); and the length of time since last routine checkup (within the past year versus 12 months or longer). We acknowledge the importance of including gender identity and racial and ethnic subgroups individually; however, cell sizes in the 2020 data were not robust enough for the intended analyses.

### Data Analyses

To describe the sample characteristics and outcomes across time periods, we calculated unweighted frequencies and population-weighted percentages accounting for the complex sampling design. We ran a series of Rao-Scott Chi-Square tests designed for complex survey data to test for differences across time periods in prevalence of ACEs recollections and mental health days. We calculated the change in population-reported ACEs recollections and mental health days by subtracting the weighted percentages across time periods.

We tested the moderation effect of our sample characteristics on change in population-reported outcomes across time periods. We ran moderated multiple logistic regression for binary outcomes and moderated multiple ordered logistic regression for ordinal outcomes to test for significant interactions of subgroups by time period (Hayes, 2013). The model included time period, the 13 sample characteristics, and their interactions with the time period. If the interaction was significant, we ran a series of simple effects tests to investigate if there is an effect of the sample characteristics on the difference in the outcome from before to after COVID-19 began (Hayes, 2013). We accounted for the complex sampling design in the modeling.

We conducted all data management and analyses in SAS version 9.4. We considered statistical tests significant at alpha ≤ 0.05. Given these are publicly available data, this study was confirmed by the University of Missouri Institutional Review Board that it does not meet federal definitions for human subjects research, and therefore no institutional review board approval was required.

## Results

### Descriptive Characteristics

Our sample includes 45,198 records for participants with at least one non-missing outcome variable across 2019 and 2020 (Fig. 1). Sample characteristics are provided in Table 1.

**Table 1:** Sample characteristics of 2019 Behavioral Risk Factor Surveillance System data for 17 U.S. States implementing the Adverse Childhood Experiences module

	Before COVID-19 Started January—April 2019		After COVID-19 Started January—April 2020	
	No.^a^	Weighted % (95% CI)	No.^a^	Weighted % (95%CI)
	39,358	84.6 (84.0–85.1)	5840	15.5 (14.9–16.0)
Sex				
Male	17,605	48.9 (48.0–49.8)	2623	48.5 (46.5–50.6)
Female	21,753	51.1 (50.2–52.0)	3217	51.5 (49.4–53.5)
Age group				
18–34	6229	28.2 (27.3–29.0)	995	28.9 (26.8–31.0)
35–54	10,012	31.5 (30.6–32.4)	1566	32.7 (30.7–34.7)
55–64	8208	17.3 (16.6–17.9)	1172	17.3 (16.0–18.6)
65+	14,909	23.1 (22.5–23.8)	2107	21.1 (19.7–22.4)
Race/ethnicity				
Non-Hispanic White	30,441	71.6 (70.7–72.5)	4442	70.6 (68.5–72.7)
Other	8156	28.4 (27.5–29.3)	1293	29.4 (27.3–31.5)
Education				
Did not graduate High School	3135	12.0 (11.3–12.7)	348	9.3 (8.0–10.7)
Graduated High School	11,478	31.0 (30.2–31.8)	1629	32.0 (30.1–33.8)
Attended College or Technical School	10,949	31.0 (30.2–31.9)	1640	30.1 (28.2–32.0)
Graduated from College or Technical School	13,632	26.0 (25.3–26.7)	2205	28.6 (26.8–30.5)
Employment				
Employed/self-employed	18,388	55.4 (54.5–56.3)	2874	57.8 (55.8–59.8)
Retired	12,613	21.3 (20.6–22.0)	1746	18.8 (17.6–20.1)
Other (out of work, homemaker, student, unable to work)	7811	23.3 (22.5–24.1)	1133	23.3 (21.5–25.2)
Annual household income				
< $25,000	8714	25.9 (25.1–26.8)	1156	22.9 (21.1–24.6)
$25,000 to < $50,000	8004	24.2 (23.4–25.1)	1184	23.5 (21.7–25.3)
$50,000 or more	15,367	49.8 (48.9–50.8)	2414	53.7 (51.5–55.9)
Own, rent, or other living arrangement				
Own	28,431	70.5 (69.6–71.3)	4236	71.4 (69.5–73.2)
Rent/other arrangement	10,623	29.5 (28.7–30.4)	1559	28.6 (26.8–30.5)
Rural/urban designation				
Rural	6557	9.59 (9.2–10.0)	719	6.7 (5.9–7.4)
Urban	32,801	90.4 (90.0–90.8)	5121	93.3 (92.6–94.1)
Married/partnered				
Yes	21,040	55.4 (54.5–56.3)	3067	54.9 (52.9–56.9)
No	18,001	44.6 (43.7–45.5)	2740	45.1 (43.1–47.1)
Number of adults in household (ages 18 and older)				
One	12,713	23.9 (23.2–24.6)	1930	25.0 (23.4–26.6)
Two	19,277	50.0 (49.0–50.9)	2775	48.7 (46.7–50.7)
Three or more	7266	26.2 (25.3–27.0)	1120	26.3 (24.4–28.2)
Children in household (< age 18)				
Any	9537	33.3 (32.4–34.2)	1467	34.9 (32.9–37.0)
None	29,129	66.7 (65.8–67.6)	4282	65.1 (63.0–67.1)
Health care coverage				
Yes	35,941	88.6 (88.0–89.3)	5314	89.2 (88.0–90.4)
No	3226	11.4 (10.7–12.0)	490	10.8 (9.6–12.0)
Time since last routine checkup				
Within past year	32,019	77.3 (76.5–78.1)	4740	78.6 (76.7–80.4)
12 months or longer	7335	22.7 (21.9–23.5)	1100	21.4 (19.6–23.3)

^a^Unweighted

### Prevalence of Poor Mental Health Days and ACEs

Table 2 reports the prevalence of the outcomes, overall and by time period. Overall, 65.3% of US adults reported one or more ACEs, and 37.3% reported one or more poor mental health days in the past 30 days. Emotional abuse is the most prevalent ACE reported across the 17 states (35.0%).

**Table 2:** Prevalence, Change in Percent, and Unadjusted Change in the Odds of Recalled Adverse Childhood Experiences (ACE) and Reported Mental Health Status, Overall and by Time Period, 2019 BRFSS Data

	Overall n = 45,198		Before COVID-19 Started Jan-Apr 2019 n = 39,358		After COVID-19 Started Jan-Apr 2020 n = 5840		*P* value^c^	Change in Percent from 2019 to 2020	Crude Odds Ratio
	No.^a^	Weighted % (95% CI)	No.^a^	Weighted % (95% CI)	No.^a^	Weighted % (95% CI)			
ACEs recollections									
Mental illness	6154	18.5 (17.8–19.2)	5294	18.1 (17.4–18.9)	860	20.5 (18.8–22.2)	**.01**	2.39	**1.17 (1.04–1.31)**
Substance abuse	10,067	28.4 (27.6–29.2)	8817	28.3 (27.4–29.1)	1250	29.4 (27.4–31.3)	.29	1.14	1.06 (0.95–1.17)
Incarcerated household member	2576	9.7 (9.0–10.3)	2242	9.8 (9.09–10.4)	334	9.0 (7.8–10.2)	.29	− 0.78	0.91 (0.77–1.08)
Parental separation or divorce	9241	30.7 (29.8–31.5)	8102	30.7 (29.7–31.6)	1139	30.7 (28.6–32.7)	.99	− 0.01	1.00 (0.90–1.11)
Intimate partner violence	5891	17.5 (16.8 -18.2)	5148	17.4 (16.6–18.1)	743	18.1 (16.5–19.7)	.43	0.72	1.05 (0.93–1.19)
Physical abuse	8638	24.4 (23.7–25.2)	7521	24.1 (23.3–24.9)	1117	26.3 (24.4–28.2)	**.03**	2.18	**1.12 (1.01–1.25)**
Emotional abuse	11,800	35.0 (34.2–35.9)	10,233	34.7 (33.7–35.6)	1567	37.0 (35.0–39.1)	**.04**	2.36	**1.11 (1.01–1.22)**
Sexual abuse	4725	13.0 (12.4–13.5)	4096	12.6 (12.0–13.3)	629	14.9 (13.4–16.4)	**.004**	2.27	**1.21 (1.06–1.38)**
Sum of recalled ACEs^b^									
0	14,899	34.7 (33.9–35.6)	13,152	35.2 (34.3–36.1)	1747	32.0 (30.1–34.0)	**.04**	− 3.20	**1.11 (1.03–1.21)**
1	9024	23.6 (22.8–24.3)	7895	23.4 (22.6–24.3)	1129	24.2 (22.3–26.0)		0.74	
2 or 3	8502	23.2 (22.5–24.0)	7405	23.0 (22.2–23.8)	1097	24.6 (22.8–26.4)		1.62	
4+	5830	18.5 (17.8–19.2)	5073	18.4 (17.6–19.1)	757	19.2 (17.5–20.9)		0.84	
Days during the past 30 when mental health not good^b^									
Zero	29,104	62.7 (61.9–63.5)	25,498	63.1 (62.2–64.0)	3606	60.4 (58.3–62.5)	**.03**	− 2.70	**1.10 (1.01–1.20)**
1–13	9471	23.5 (22.7–24.2)	8105	23.1 (22.3–23.9)	1366	25.6 (23.7–27.5)		2.52	
14+	5647	13.8 (13.3–14.4)	4902	13.8 (13.2–14.4)	745	14.0 (12.6–15.3)		0.19	

^a^Unweighted

^**b**^Reported odds ratio represents increase in odds to score higher on response

^c^Weighted Rao-Scott Chi-Square

Bold values indicate *p* < 0.05

### Increases in Prevalence of Poor Mental Health Days and ACEs, Before Compared to After COVID-19 Began

We found a significant increase (+ 2.70%) from before compared to after COVID-19 began in the prevalence of reported poor mental health days in the past 30 days. The crude odds of reporting more poor mental health days is 1.10 higher after COVID-19 began compared to before.

We found a significant change from before compared to after COVID-19 began in the prevalence of reported ACEs for the following: living with someone with mental illness (+ 2.39%), physical abuse (+ 2.18%), emotional abuse (+ 2.36%), sexual abuse (+ 2.27%), and sum of ACEs (-3.20% for no ACEs). The crude odds of reporting the following ACEs are significantly higher after compared to before COVID-19 began: living with someone with mental illness by 1.17 times; having an adult in the home physically abuse you in any way by 1.12 times; having an adult ever emotionally abuse you by 1.11 times; being sexually abused by 1.21 times; and a higher sum of ACEs by 1.11 times.

### Increases in Prevalence of Poor Mental Health Days and ACEs, Before Compared to After COVID-19 Began, by Subgroups

The following interactions between subgroups and time period were significant in the moderated logistic and ordered logistic models (adjusted models): race/ethnicity for mental illness (Type III F statistic and p-value: F(1,30476) = 4.38 (p = 0.036)); race/ethnicity for substance abuse (F(1,30538) = 11.5 (p = 0.0007)); employment for sexual abuse (F(2,30163) = 4.37 (p = 0.013)); race/ethnicity for sum of ACEs (F(1,30571) = 6.23 (p = 0.013)); and marital status for poor mental health days (F(1,30163) = 9.60 (p = 0.002)).

Table 3 displays the significant interactions from the adjusted logistic regression models of subgroups by time period. The adjusted odds for those married or partnered reporting more poor mental health days in the past 30 days was 1.48 times higher after the pandemic started compared to before. No such effect was observed for those divorced, widowed, separated or never married. Regarding ACEs, the adjusted odds for the prevalence of POC reporting living with anyone with mental illness during childhood was 1.73 times higher after the pandemic started compared to before. No such effect was observed for Non-Hispanic Whites. The adjusted odds for the prevalence of those employed or self-employed reporting childhood sexual abuse was 1.45 times higher after the pandemic started compared to before; this effect was borderline significant. No such effect was observed for those retired, out of work, homemaker, student, or unable to work. Finally, the adjusted odds for the prevalence of POC reporting more ACEs was 1.35 times higher after the pandemic started compared to before. No such effect was observed for non-Hispanic Whites.

**Table 3:** Significant Simple Effects of Before and After COVID-19 Periods at Levels of Sample Characteristics on ACEs Recollections and Mental Health Reporting, Reported Only for Significant Interactions in the Moderated Logistic or Ordered Logistic Model, 2019 BRFSS Data

	Sample characteristics level	Estimated Mean Change in Log Odds from Pre- to Early-Onset Pandemic (Std Err)	t-statistic	Odds Ratio (95% CI)^a^	*P* value
ACEs recollections					
Mental illness	Race/ethnicity other than non-Hispanic White	0.55 (.209)	t(30,476) = 2.62	**1.73 (1.15, 2.61)**	**0.009**
Substance abuse	None significant				
Incarcerated household member	None significant				
Parental separation/divorce	None significant				
Intimate partner violence	None significant				
Physical abuse	None significant				
Emotional abuse	None significant				
Sexual abuse	Employed/self-employed	0.37 (.187)	t(30,163) = 1.98	**1.45 (1.00, 2.08)**	**0.048**
Sum of recalled ACEs	Race/ethnicity other than non-Hispanic White	0.30 (.146)	t(30,571) = 2.07	**1.35 (1.02, 1.81**)	**0.038**
0					
1					
2 or 3					
4+					
Days during the past 30 when mental health not good	Married/partnered	0.39 (0.144)	t(30,163) = 1.98	**1.48 (1.12, 1.96)**	**0.006**
Zero					
1–13					
14+					

^a^The model included time period, the 13 sample characteristics, and their interactions with the time period

Bold values indicate *p* < 0.05

## Discussion

Our study identified significant increases in population-level prevalence of a robust number of psychological burden indicators (poor mental health days in the past 30 days and recollections of childhood exposures to mental illness, physical abuse, emotional abuse, and sexual abuse, and sum of ACEs) before compared to after the onset of the pandemic among U.S. adults in 17 states. Based on crude odds ratios, post-pandemic respondents were ten to seventeen percent more likely to affirm a measure of psychological burden compared to those interviewed before the pandemic.

Our study revealed the prevalence of those married or partnered reporting more poor mental health days in the past 30 days was 48% higher after the pandemic started compared to before. Why no such effect was observed for those divorced, widowed, separated, or never married may be related to within-household *coordination* of daily routines from school closures, job loss, and income insecurity that may be absent from a single head of household.

The prevalence of POC reporting living with anyone with mental illness during childhood was 73% more likely, and reporting more ACEs was 35% more likely after the pandemic started compared to before. We offer several interpretations for the disproportionate change in population-level prevalence from communities of color that was not observed among non-Hispanic Whites. The increased reporting of exposure to mental illness in childhood and multiple ACEs may be due to population-level changes in perceived threats and experiences that influence reporting, such as anti-Asian harassment and xenophobia that began as a result of the COVID-19 outbreak’s reported origins in Wuhan, China (Gover et al., 2020). Over a four week period from March to April 2020, 1,497 reports of COVID-19-related discrimination occurred against Asian American and Pacific Islanders (Jeung, 2020). Moreover, Black and Hispanic U.S. populations experienced higher rates of infection and COVID-19-related mortality compared to non-Hispanic Whites (Mackey et al., 2021). As stated earlier, an association has been reported that those with ACEs are more likely to develop anxiety disorders and perceive a greater threat from COVID-19 (Kalia et al., 2020), and persons of color are more likely to have more ACE exposures (Merrick et al., 2018, 2019). Finally, highly public anti-Black violence co-occurred during this time frame, including to Ahmaud Arbery who was fatally shot while jogging in Glynn County, Georgia on February 23, 2020, and Breonna Taylor who was killed in her home on March 13, 2020. This violence has been associated with poor mental health days among Black Americans (Curtis et al., 2021).

Finally, our study observed the prevalence of those employed or self-employed reporting childhood sexual abuse was 45% higher after the pandemic started compared to before. While the confidence interval includes the null value of 1.0, we anticipate the effect would have been stronger with an increased cell size given the adjusted model and interaction term (i.e. 629 reported childhood sexual abuse in 2020). With no such effect observed for those retired, out of work, homemaker, student, or unable to work, these findings may be related to interpersonal interactions at work that may have heightened reflection or reporting. Regarding implications, sexual abuse ACEs are independently associated with negative health outcomes, including smoking, risky HIV behavior, obesity, diabetes, coronary heart disease, depression, and disability caused by poor health (Campbell et al., 2016).

The BRFSS is a crucial surveillance tool sponsored by the CDC and used for measuring the changes in the health of the U.S. population. As a weighted population-based study, the results are generalizable to the U.S. population. Regarding limitations, the most important limitation to underscore is the cross-sectional nature of the BRFSS, which precludes the ability to make causal inferences. Specifically, the BRFSS ACEs questions are designed to capture adult reflections of their exposures younger than age 18; thus, findings do not reflect incidence (i.e. newly emergent ACEs from the pandemic). These are ecological associations across multiple populations and time points, and therefore subject to history effects, recall, and reporting bias. Despite these cautions in interpretation, the value of ecological-level analyses from the BRFSS is their appropriateness as first steps in identifying population-level differences, their ability to generalize to the population, and their robust sample size. We observed significant changes in the prevalence of psychological burden reporting for population subgroups that may reflect heightened, accentuated, or exasperated present-day circumstances, and thus changes in self-reflection and/or willingness to report, given the extraordinary events of these times. We encourage additional study in this area. Finally, the BRFSS captures data on the original ACEs definitions given the large body of supporting research (Centers for Disease Control & Prevention, 2020); however, emerging research has advocated for an expanded definition, including community dysfunction, bullying, and property victimization (Karatekin & Hill, 2019).

This is the first study to examine prevalence of poor mental health days and ACEs recollections in US adults before and after COVID-19 began. Supportive recovery from the COVID-19 pandemic needs to emphasize accessible, equitable, effective, and culturally responsive mental health services. Without such support, many adults could experience the long-term and deleterious effects of untreated psychological distress.
